# Fighting Diseases

**Published:** 1916-05

**Authors:** 


### Fighting Diseases.

Texas health authorities, in connection with the International Health Commission and the Extension Department of the University of Texas ,are making an organized and systematic effort to stamp out malaria, hookworm, typhoid and dysentery in certain infected sections in East Texas. Within the areas in which operations are being conducted every family has agreed to submit to all the regulations required and to follow all recommendations. If these experiments are successful, and there is every reason for believing they will at least prove their value, other sections of the State and of the South will likely profit by them. Not only physicians but the laity everywhere will watch the results of this campaign with much interest.

